# A Novel Non-Lens βγ−Crystallin and Trefoil Factor Complex from Amphibian Skin and Its Functional Implications

**DOI:** 10.1371/journal.pone.0001770

**Published:** 2008-03-12

**Authors:** Shu-Bai Liu, Ying-Ying He, Yun Zhang, Wen-Hui Lee, Jin-Qiao Qian, Ren Lai, Yang Jin

**Affiliations:** 1 Biotoxin Units, Key Laboratory of Animal Models and Human Disease Mechanisms, Kunming Institute of Zoology, The Chinese Academy of Sciences, Kunming, Yunnan, China; 2 Graduate School of the Chinese Academy of Sciences, Beijing, China; National Institutes of Health, United States of America

## Abstract

**Background:**

In vertebrates, non-lens βγ-crystallins are widely expressed in various tissues, but their functions are unknown. The molecular mechanisms of trefoil factors, initiators of mucosal healing and being greatly involved in tumorigenesis, have remained elusive.

**Principal Findings:**

A naturally existing 72-kDa complex of non-lens βγ-crystallin (α-subunit) and trefoil factor (β-subunit), named βγ-CAT, was identified from frog *Bombina maxima* skin secretions. Its α-subunit and β-subunit (containing three trefoil factor domains), with a non-covalently linked form of αβ_2_, show significant sequence homology to ep37 proteins, a group of non-lens βγ-crystallins identified in newt *Cynops pyrrhogaster* and mammalian trefoil factors, respectively. βγ-CAT showed potent hemolytic activity on mammalian erythrocytes. The specific antiserum against each subunit was able to neutralize its hemolytic activity, indicating that the two subunits are functionally associated. βγ-CAT formed membrane pores with a functional diameter about 2.0 nm, leading to K^+^ efflux and colloid-osmotic hemolysis. High molecular weight SDS-stable oligomers (>240-kDa) were detected by antibodies against the α-subunit with Western blotting. Furthermore, βγ-CAT showed multiple cellular effects on human umbilical vein endothelial cells. Low dosages of βγ-CAT (25–50 *p*M) were able to stimulate cell migration and wound healing. At high concentrations, it induced cell detachment (EC_50_ 10 nM) and apoptosis. βγ-CAT was rapidly endocytosed via intracellular vacuole formation. Under confocal microscope, some of the vacuoles were translocated to nucleus and partially fused with nuclear membrane. Bafilomycin A1 (a specific inhibitor of the vacuolar-type ATPase) and nocodazole (an agent of microtuble depolymerizing), while inhibited βγ-CAT induced vacuole formation, significantly inhibited βγ-CAT induced cell detachment, suggesting that βγ-CAT endocytosis is important for its activities.

**Conclusions/Significance:**

These findings illustrate novel cellular functions of non-lens βγ-cyrstallins and action mechanism via association with trefoil factors, serving as clues for investigating the possible occurrence of similar molecules and action mechanisms in mammals.

## Introduction

In vertebrates, crystallins are structural proteins that define the refractive index and the optical properties of the lens tissue. Among the three ubiquitous crystallins (α, β and γ), α-crystallins are related to the small heat-shock proteins, while β- and γ-crystallins belong to the same superfamily, which also includes microbial stress-inducible proteins [1]. Proteins in βγ-crystallin superfamily contain repeats of a characteristic Greek key motif of about 40 residues and two motifs associate with pseudosymmetry to form one domain. Ep37 proteins and mammalian Absent In Melanoma 1 (AIM1) are non-lens βγ-crystallins described in vertebrates. Ep37 proteins are found in embryonic epidermis, cutaneous glands and gastric epithelial cells of amphibian *Cynops pyrrhogaster* [2]–[4], and AIM1 mRNAs of different transcript sizes are temporally regulated during embryogenesis and also found in adult skin, heart, lung, liver [5], [6]. Although participation in epidermis development and a tumor suppression function have been proposed for ep37 proteins and AIM1 gene [2]–[7], little is known about the biochemical properties, functions and action mechanisms of these non-lens βγ-crystallins in vertebrates.

The trefoil factor (TFF) proteins are secreted proteins that are characterized by a conserved motif known as the TFF domain (or P-domain previously). This domain consists of some 40 amino acid residues in which six cysteines are disulfide-linked in a 1–5, 2–4, and 3–6 configurations [8], [9]. Three closely related mammalian TFF proteins are known. TFF1 and TFF3 contain a single TFF domain, while TFF2 contains two TFF-domains. In amphibian, a molecular cloning approach revealed the existence of a two TFF-domain protein xP2 in *Xenopus laevis* skin, and TFFs containing single TFF-domain (xP1) and four TFF-domains (xP4) were also cloned from the stomach of *X. laevis* [10], [11]. Furthermore, a two TFF-domain protein (Bm-TFF2) that shows human platelet activation activity has been reported from frog *Bombina maxiam* skin previously [12].

In mammals, the main expression of TFFs are found in specific epithelial cells of the gastrointestinal tract, in which they play an important role in the repair and healing of the gastrointestinal tract by stimulating the migration of cells at the mucosal wounding edges [13], [14]. TFFs are also found in many cancers, and TFF1 knock-out mice exhibit tumor increasing, indicating that it may be a specific tumor suppressor gene for stomach [15], [16]. In addition, TFFs have been proposed as inflammatory mediators and connected with a possible role in immune regulation [14], [17]. However, key questions remain to be resolved to achieve a full understanding of the first-hand actions of TFFs and the molecular mechanisms involved [14].

The Chinese red belly frog (*B. maxima*) is an endemic amphibian in the mountainous regions of southwestern China. It has been known by the indigenous people that the frog lives in very harsh environments and its skin is very “toxic”. Here we show the purification, molecular cloning and functional characterization of a novel protein that is responsible for the potent hemolytic activity and lethal toxicity on mice of the frog skin secretions. We demonstrated that this protein is a naturally existing complex of non-lens βγ-crystallin and trefoil factor, named βγ-CAT. βγ-CAT caused hemolysis of mammalian erythrocytes via membrane pore formation. On the other hand, it was able to stimulate cell migration and wound healing, as well as induce cell detachment and cell apoptosis depending on the dosages used, as assayed in human umbilical vein endothelial cells (HUVECs). Rapid endocytosis of βγ-CAT via intracellular vacuole formation and potential translocation to cell nucleus were also observed. For the first time, our findings demonstrate the combination of a non-lens βγ-crystallin member and a trefoil factor, and the potent capacity resulted in the regulation of cell migration, survival and apoptosis.

## Results

### Purification of βγ-CAT

**Figure 1:**
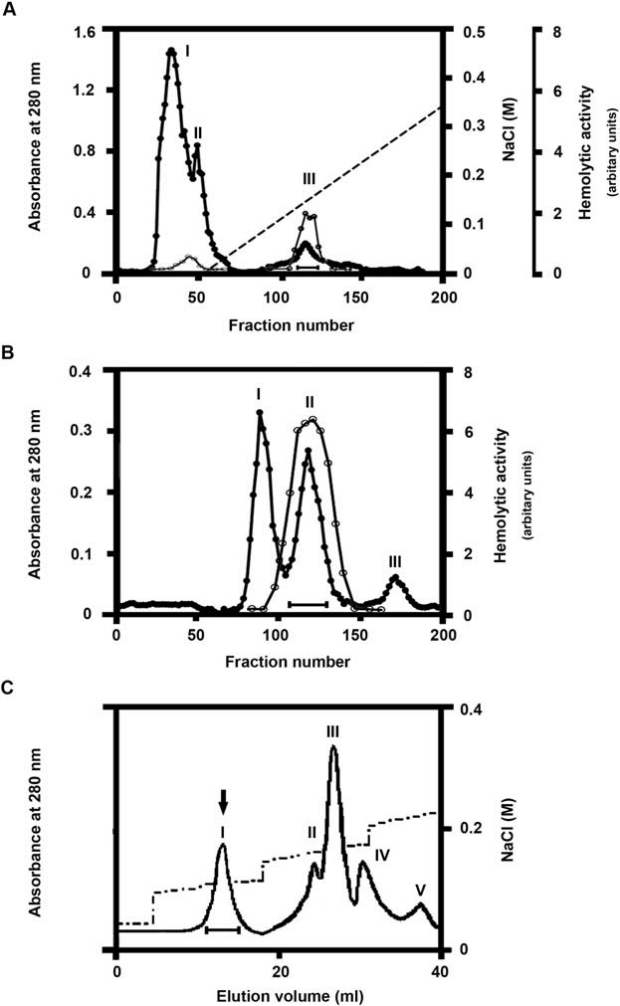
Purification of βγ-CAT. (A) Lyophilized frog *B. maxima* skin secretions (5.0 g, containing 550 mg proteins, from a stock in Kunming Institute of Zoology) was dissolved in 10 ml 50 mM Tris-HCl buffer, pH 7.3, containing 5 mM EDTA, dialyzed against the same buffer at 4°C overnight and centrifuged. The supernatant was loaded on a DEAE Sephadex A-50 (Pharmacia) column (2.6×50 cm). The elution was performed at a flow rate of 30 ml/h with a linear NaCl gradient, collecting fractions of 5 ml per tube. Hemolytic activity was mainly found in NaCl-eluted peak III. This peak was collected as indicated by a bar. (B) The peak III from DEAE Sephadex A-50 column was concentrated, then applied on a Sephadex G-100 (Pharmacia, superfine) column (2.6×100 cm) equilibrated with 50 mM Tris-HCl buffer, pH 7.8, containing 150 mM NaCl and 5 mM EDTA. Elution was achieved with the same buffer at a flow rate of 12 ml/h, collecting fractions of 2 ml per tube. The hemolytic activity was found in peak II. In A and B, The protein concentration was estimated from the absorbance at 280 nm (•). The hemolytic activity on human erythrocytes (○) was determined as described in “Methods”. (C) The peak II of Sephadex G-100 column was lyophilized and dialyzed against 25 mM Tris-HCl, pH 7.8, for 24 h at 4°C, and finally applied on an AKTA Mono-Q HR5/5 anion ion exchange column equilibrated with the same buffer. Elution was performed at a flow rate of 1.0 ml/min with the NaCl gradient, as shown in the figure. Hemolytic activity was found in peaks I, II and III. Peak I is highly purified βγ-CAT, as indicated by an arrow.

Each step of the purification was followed by determining hemolytic activity on human erythrocytes for each fraction. Initiated with 5.0 g of lyophilized *B. maxima* skin secretions (containing 550 mg proteins), DEAE Sephadex A-50 column at pH 7.3 resulted in separation of three protein peaks, in which hemolytic activity was predominantly associated with NaCl-eluted peak III (Fig. 1A). In addition to antimicrobial and mytropic activities caused by antimicrobial peptides and Bv8 analogous [18], [19], platelet activation activity was found in NaCl-free eluted peaks I and II, from which Bm-TFF2 has been purified previously [12]. Subsequent gel filtration of DEAE-Sephadex A-50 column peak III resulted in separation of three protein peaks, in which hemolytic activity was found in peak II (Fig. 1B). The peak II of Sephadex G-100 column was collected and finally applied on an AKTA Mono-Q HR5/5 anion ion exchange column. This purification step resulted in separation of several protein peaks, in which peak I is highly purified βγ-CAT associated again with hemolytic activity (Fig. 1C). Finally, about 650 µg product was obtained. Purified βγ-CAT showed lethal toxicity on mice. The LD_50_ values were 20 µg/kg and 400 µg/kg under intravenous and intraperitoneal injections, respectively. Purified protein showed neither proteolytic nor phospholipase A_2_ activity. Hemolytic activity was also determined in Peaks II and III of AKTA Mono-Q HR5/5 column, but these fractions lacked lethal toxicity on mice (LD_50_ value>5 mg/kg under intraperitoneal injection) and native polyacrylamide gel electrophoresis (PAGE) and SDS-PAGE analysis revealed their heterogeneity.

### Characterization of βγ-CAT

**Figure 2:**
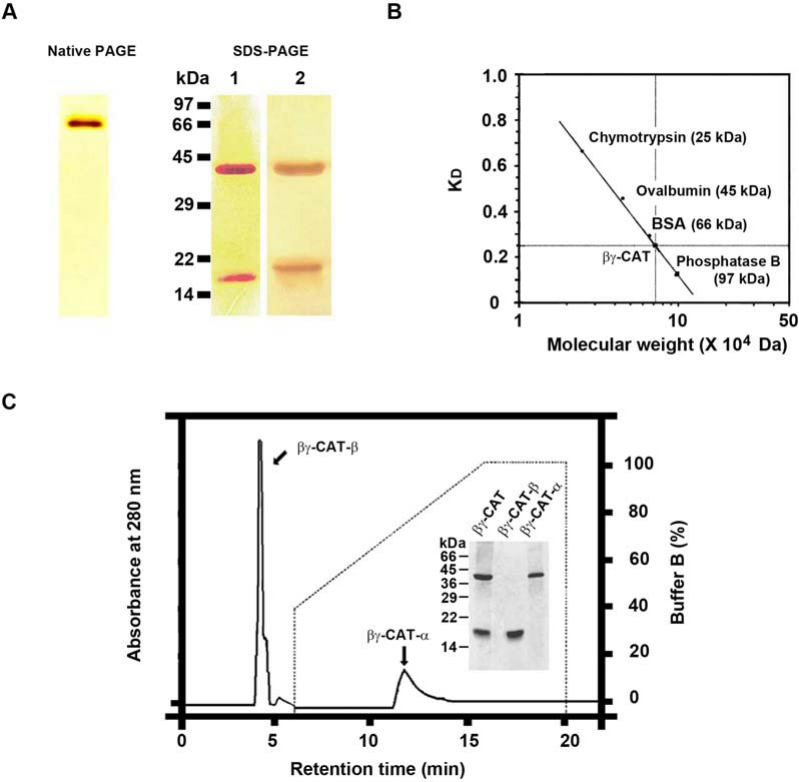
Characterization of βγ-CAT. (A) PAGE analysis of purified βγ-CAT. Left part, PAGE analysis of purified βγ-CAT under native conditions at pH 8.5 (10% acrylamide). Right part, SDS-PAGE (10% acrylamide) analysis of purified βγ-CAT under reducing conditions (lane 1) and non-reducing conditions (lane 2). The protein bands were silver-stained. (B) Native molecular weight analysis of βγ-CAT by gel filtration on a Sephadex G-100 column. (C) HPLC isolation of βγ-CAT subunits. Purified βγ-CAT was loaded on a Vydac C_4_ reverse phase HPLC column equilibrated with 30% (v/v) acetonitrile/water containing 0.1% trifluoroacetic acid. The elution was performed with a gradient of buffer B (30% isopropanol plus 70% acetonitrile, containing 0.1% trifluoroacetic acid) at a flow rate of 1 ml/min. Insert, SDS-PAGE (10% acrylamide, under reducing conditions) analysis of isolated βγ-CAT subunits.

Purified protein migrated as a single band in native-PAGE with silver staining. However, in SDS-PAGE under both reducing and non-reducing conditions, the protein was separated into two bands with apparent molecular weights of 38,000 Da (α-subunit) and 18,000 Da (β-subunit). The protein bands were analyzed by Gel-Pro analyzer software. The α- and β-subunits accounted for 56% and 44% of the total protein, respectively (Fig. 2A). Since the molecular weight of the α-subunit is about 2-fold of that of the β-subunit, the approximately equal protein percentage of each subunit in the molecule suggested that the molecular ratio of the two subunits is of 1∶2. On the other hand, gel filtration analysis revealed that the apparent molecular weight of native βγ-CAT was 72,000 Da (Fig. 2B). Taken together, it was suggested that βγ-CAT is composed of two types of subunits connected non-covalently with a structure of αβ_2_. The β-subunit was readily isolated in a HPLC C_4_ column and eluted with 30% acetonitrile. In contrast, the α-subunit appeared to be highly hydrophobic and was difficult to be eluted and obtained in the HPLC column (Fig. 2C).

### Molecular cloning of βγ-CAT

Each subunit of βγ-CAT was subjected to amino acid sequencing by Edman degradation. The N-terminal 15 amino acids of the β-subunit were determined. In addition, the sequences of several internal peptides (accounting to 58 amino acid residues) were also obtained. Repeated effort to sequence the α-subunit was unsuccessful, indicating that its N-terminus is blocked. On the basis of obtaining full β-subunit sequence, six internal peptide sequences (accounting to 98 amino acid residues) of the α-subunit were thus acquired by trypsin digestion of native βγ-CAT. Partial cDNA sequences obtained by PCR amplifications with degenerated primers designed according to obtained βγ-CAT peptide sequences of each subunit greatly facilitated subsequent molecular cloning. A cDNA library constructed from *B. maxima* skin was screened at high stringency by an efficient and rapid PCR-based procedure [18]. Positive clones were identified and isolated. Both strands of the clone were sequenced.

**Figure 3:**
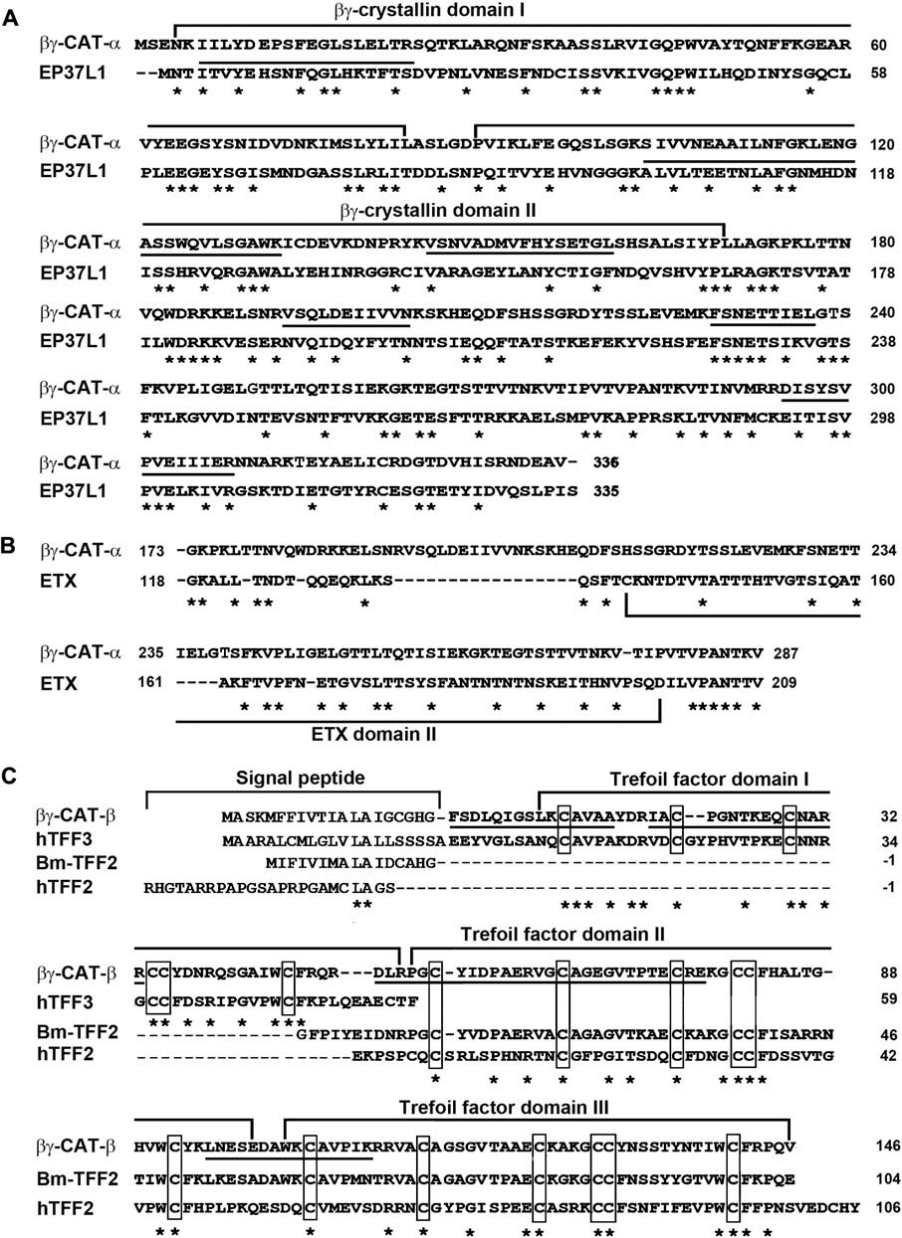
Sequence comparison of βγ-CAT α- and β-subunits. (A) Sequence comparison of βγ-CAT α-subunit with EP37L1 from newt *C. pyrrhogaster* [2]. The characteristic βγ-crystallin domains were marked. (B) Sequence comparison of βγ-CAT α-subunit C-terminal part (residues 173–287) with an internal fragment (residues 118–209) of ETX from bacterial *C. perfringens* [21]. (C) Sequence comparison of βγ-CAT β-subunit with human TFF3 [22], Bm-TFF2 [12] and human TFF2 [23]. The characteristic cysteine residues of TFF domains are boxed. In (A) and (C), Peptides corresponding to determined amino acid sequences by Edman degradation are underlined. In (A), (B) and (C), identical residues are shown by asterisks. Gaps have been introduced to optimize the sequence homology.

The full protein sequence of the α-subunit, as deduced from its coding cDNA (data deposited in GenBank, EU003881), is composed of 336 amino acids. The determined internal amino acid sequences were found exactly in the deduced sequence (Fig. 3A, underlined), thereby unequivocally confirming the identity of the obtained cDNA clone. A BLAST search in databases revealed that the α-subunit is homologous to ep37 proteins, which are non-lens βγ-crystallin members identified in various tissues of newt *C. pyrrhogaster* [2]–[4], [20]. Its sequence shows 32% identity with that of EP37L1 (also called EDSP, epidermic differentiation specific protein). In addition, the sequence identity between its βγ-crystallin domains and human AIM1 βγ-crystallin domains 5–6 is 28%. The sequence of the α-subunit actually consists of two distinct parts (Fig. 3A and B). Similar to EP37L1, it contains four consecutive Greek key motifs organized into two βγ-crystallin domains in its N-terminal part (residues 1–170). The C-terminal part (residues 173–287) shows the most homology (sequence identity 24%) to an internal fragment (residues 118–209) of epsilon toxin (ETX) from bacterial *Clostridium perfringens* [21] (Fig. 3B).

The cDNA cloning of the β-subunit and sequence determination (GenBank, EU003882) established that the mature β-subunit is composed of 146 amino acids organized into three internally homologous TFF domains (Fig. 3C). Remarkably, its first TFF domain is highly homologous to human TFF3 [22], with a sequence identity of 50% in cysteine containing region. The sequence of its domains 2–3 exhibits 30% and 69% identities with those of human TFF2 and Bm-TFF2 [23], [12].

### Hemolytic activity and membrane pore formation

**Figure 4:**
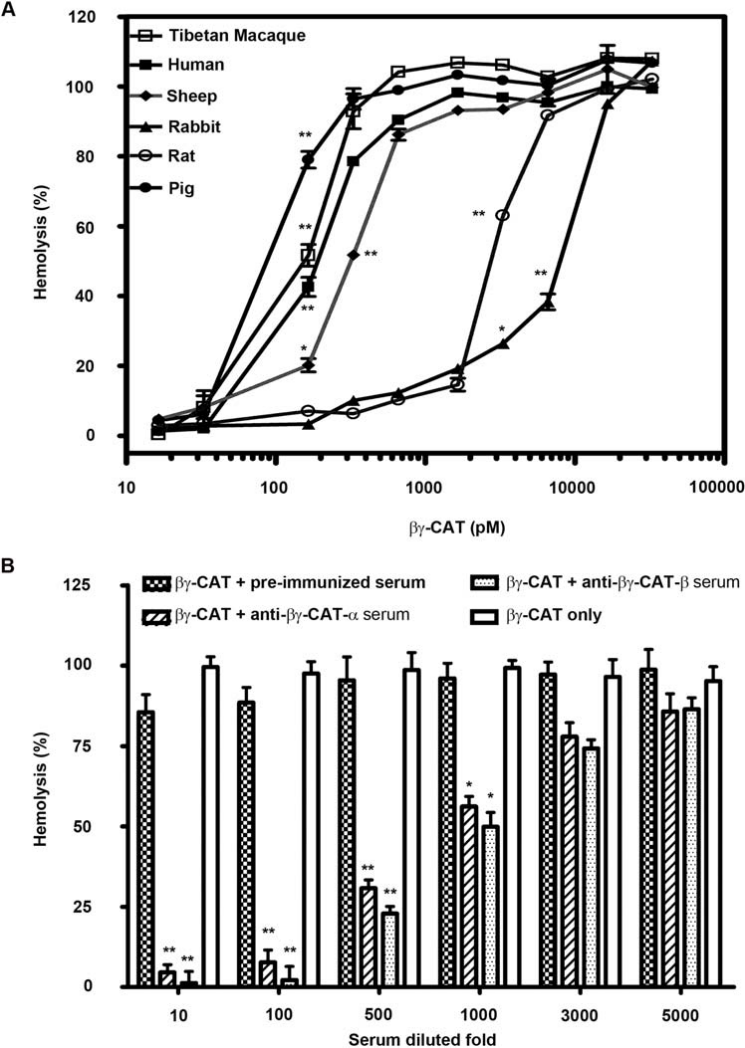
Hemolytic activity of βγ-CAT and antibody neutralization. (A) Hemolytic activity of βγ-CAT on mammalian erythrocytes. The erythrocytes (5×10^6^ cells/ml) of different species were incubated with various concentrations of βγ-CAT at 37°C for 30 min, and hemolytic activity was determined as described in “Methods” (B) Neutralization of βγ-CAT hemolytic activity by specific antiserum against each subunit of the protein. βγ-CAT (3 nM) was incubated with a series of diluted rabbit antiserum against each subunit at 37°C for 30 min, and then the rest of the hemolytic activity on human erythrocytes was determined. Pre-immunized serum was used as a negative control. The value for the erythrocytes lysed with 0.1% Triton X-100 was taken as 100%. Data were expressed as means±SEM of triplicate measurements (*, *p*<0.05; **, *p*<0.01, compared with zero βγ-CAT (A) or compared with the βγ-CAT only group (B). In (A), only initial point (*p*<0.01) was marked (**) and the following points that all have *p*<0.01 were omitted for clarity.

βγ-CAT caused hemolysis of different mammalian erythrocytes in a dose-dependent manner (Fig. 4A). The sensitivity to βγ-CAT differed among animal species. Human erythrocytes were one of the most sensitive cells, and 0.2 nM was required to induce 50% hemolysis (5×10^6^ cells/ml) (*p*<0.01, compared zero βγ-CAT). However, βγ-CAT did not show hemolytic activity on erythrocytes of king cobra snake, pigeon, duck and frog *B. maxima* (dosages used up to 30 nM). The specific antiserum against each subunit (obtained by immunizing rabbits with each purified subunit of βγ-CAT, see Methods section) was able to neutralize the hemolytic activity of βγ-CAT. 1000-fold diluted antiserum of each subunit significantly inhibited its hemolytic activity (*p*<0.05, compared with the βγ-CAT only group), while pre-immunized serum did not show inhibitory effects (Fig. 4B). HPLC purified β-subunit alone did not show hemolytic activity even with a dosage used up to 100 nM. These results indicate that the two subunits of βγ-CAT are functionally associated.

**Figure 5:**
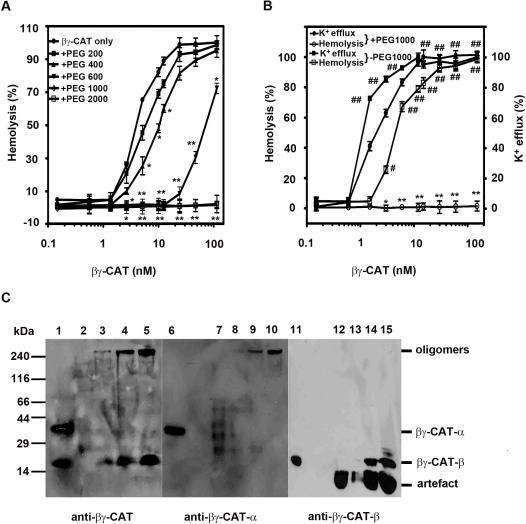
Erythrocyte membrane pore formation. (A) Osmotic protection of erythrocytes from hemolysis induced by βγ-CAT. Human erythrocytes (4×10^9^ cells/ml) were incubated with various concentrations of βγ-CAT at 37°C for 30 min in the presence of PEGs of different molecular sizes. Hemolysis was tested as described in “Methods”. (B) Human erythrocyte K^+^ efflux and hemolysis induced by βγ-CAT in the absence and presence of PEG 1000. The value of the erythrocytes lysed with 1% Triton X-100 was taken as 100%. Data were expressed as means±SEM of triplicate measurements (*, *p*<0.05; **, *p*<0.01, compared with βγ-CAT only group (A–B); #, *p*<0.05; ##, *p*<0.01, compared with zero βγ-CAT (B). (C) Western blotting analysis of SDS-stable oligomers formed in erythrocyte membranes. Human erythrocytes (6×10^7^ cells/ml) were incubated with various concentrations of βγ-CAT (0.5–3 nM) at 37°C for 30 min. Samples were treated as described in “Methods”, and loaded on a SDS-PAGE gel (linear gradient acrylamide gel of 3–15%) and blotted with rabbit polyclonal antibodies against native βγ-CAT, and each subunit, respectively. Lanes1, 6, 11, βγ-CAT control; lanes 2, 7, 12, erythrocyte control; lanes 3, 8, 13, lanes 4, 9, 14, and lanes 5, 10,15, erythrocytes treated with 0.5, 1.5, and 3 nM βγ-CAT, respectively.

K^+^ efflux was determined in human erythrocytes exposed to βγ-CAT. In βγ-CAT (3 nM) treated human erythrocytes (4×10^9^ cells/ml), about 90% of intracellular K^+^ leaked (*p*<0.01, compared with zero βγ-CAT), whereas the hemolytic rate only reached to 25% (*p*<0.05) (Fig. 5B). The erythrocyte lysis induced by βγ-CAT was also assayed in the presence of polyethylene glycols (PEGs) with different hydrodynamic diameters. The results showed that the hemolysis induced by βγ-CAT was not affected by the addition of PEGs 200, but partially inhibited by PEGs 400 and 600, and entirely suppressed by PEGs 1000 and 2000 (*p*<0.01, compared with the βγ-CAT only group) (Fig. 5A). In the presence of PEGs 1000, the hemolysis was suppressed, however, the intracellular K^+^ releasing was not obviously blocked (Fig. 5B). The hydrodynamic diameter of PEGs 1000 was estimated to be 2.0 nm [24]. These results suggest that βγ-CAT formed membrane pores with a functional diameter about 2.0 nm, leading intracellular K^+^ efflux, causing the colloid osmotic burst of erythrocytes and finally inducing erythrocyte lysis.

After treated with βγ-CAT, the erythrocyte membrane proteins were analyzed by Western blotting. Two protein bands were detected with rabbit polyclonal antibodies against native βγ-CAT. One migrated with a molecular weight greater than 240-kDa and another one showed the same molecular weight of the β-subunit (18-kDa) (Fig. 5C, left). The high molecular weight band was also recognized by specific antibodies against the α-subunit, suggesting that the α-subunit formed SDS-stable oligomers (Fig. 5C, middle). The β-subunit was not observed in the oligomers as blotted with its specific antibodies, but appeared in the same position as that of βγ-CAT control (Fig. 5C, right).

### Cellular functions

**Figure 6:**
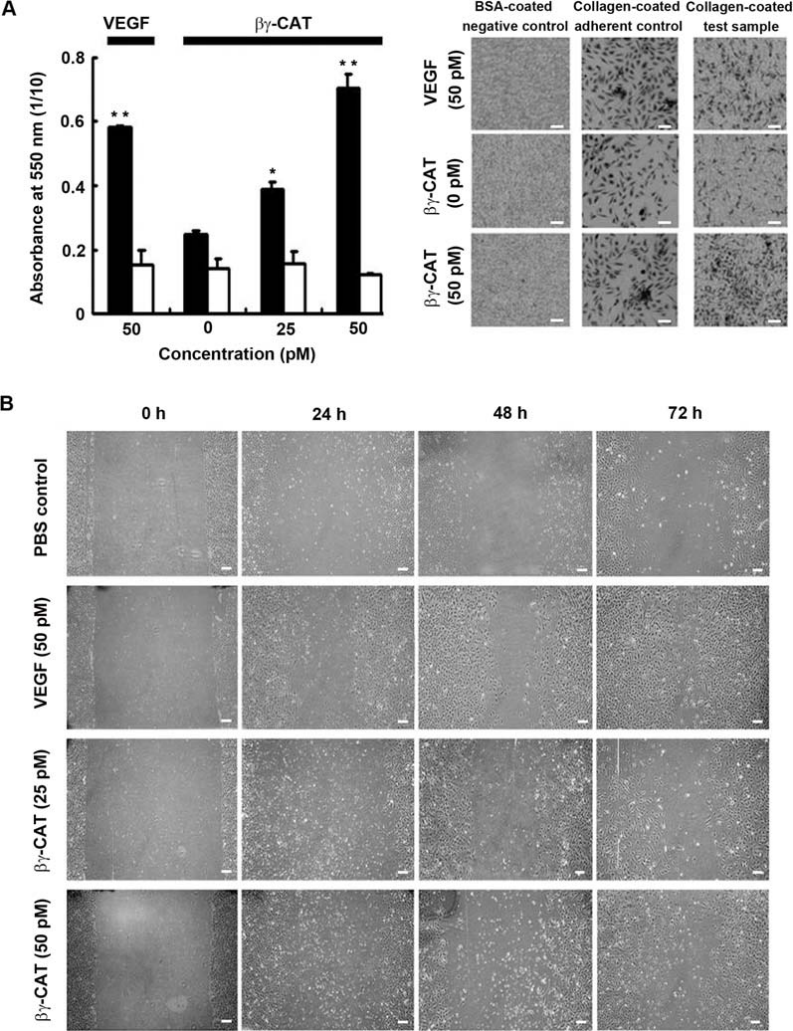
Stimulation of HUVEC migration and wound healing by low concentrations of βγ-CAT. (A) Left part, migration of HUVECs stimulated by βγ-CAT was tested by a Boyden chamber assay. Black columns and white columns present collagen-coated chambers and BSA-coated chambers, respectively. Data were expressed as means±SEM of triplicate measurements (*, *p*<0.05; **, *p*<0.01, compared with zero βγ-CAT). Right part, the recorded photography under the chambers shows obvious increase of cells after treated with βγ-CAT. (B) Closure of an artificial wound in HUVEC monolayers stimulated by βγ-CAT. The results were the representative of three independent experiments. Scale bars equal to 100 µm in (A) and (B).

βγ-CAT was initially identified and purified as a “toxic” component to mammals. It caused rabbit heart failure in an endothelium-dependent manner (Qian et al., unpublished observation). Primary cultured HUVECs were chosen for subsequent study. Since the β-subunit of βγ-CAT is a TFF, the possible activity of βγ-CAT to induce cell migration was first evaluated *in vitro*. Two assays were used. In the first assay, the ability of βγ-CAT to enhance migration in Boyden chambers was tested. Migration of HUVECs through a chamber was enhanced by βγ-CAT. HUVECs treated with βγ-CAT (25 *p*M) showed 1.5-fold the migratory activity of untreated cells (*p*<0.05, compared with zero βγ-CAT), and cells treated with βγ-CAT (50 *p*M) migrated at almost 3-fold the rate of untreated cells (*p*<0.01). Under an inverted phase-contrast microscope, increase of cells migrated through the chamber can be clearly observed in βγ-CAT treated cells (Fig. 6A). In the second assay, a defined “wound” was scraped across a HUVEC monolayer cultured in serum-starved medium on collagen. In contrast to PBS treated cells, at each time point, a slight increase in the rate of wound closure was noted in the presence of βγ-CAT (25 *p*M). At 50 *p*M, the increase was more obvious. Cells cultured in βγ-CAT (50 *p*M) achieved almost complete wound closure within 72 h (Fig. 6B). Both purified α- and β-subunits alone did not show wound healing effect with dosages used up to 30 nM (data not shown for clarity).

**Figure 7:**
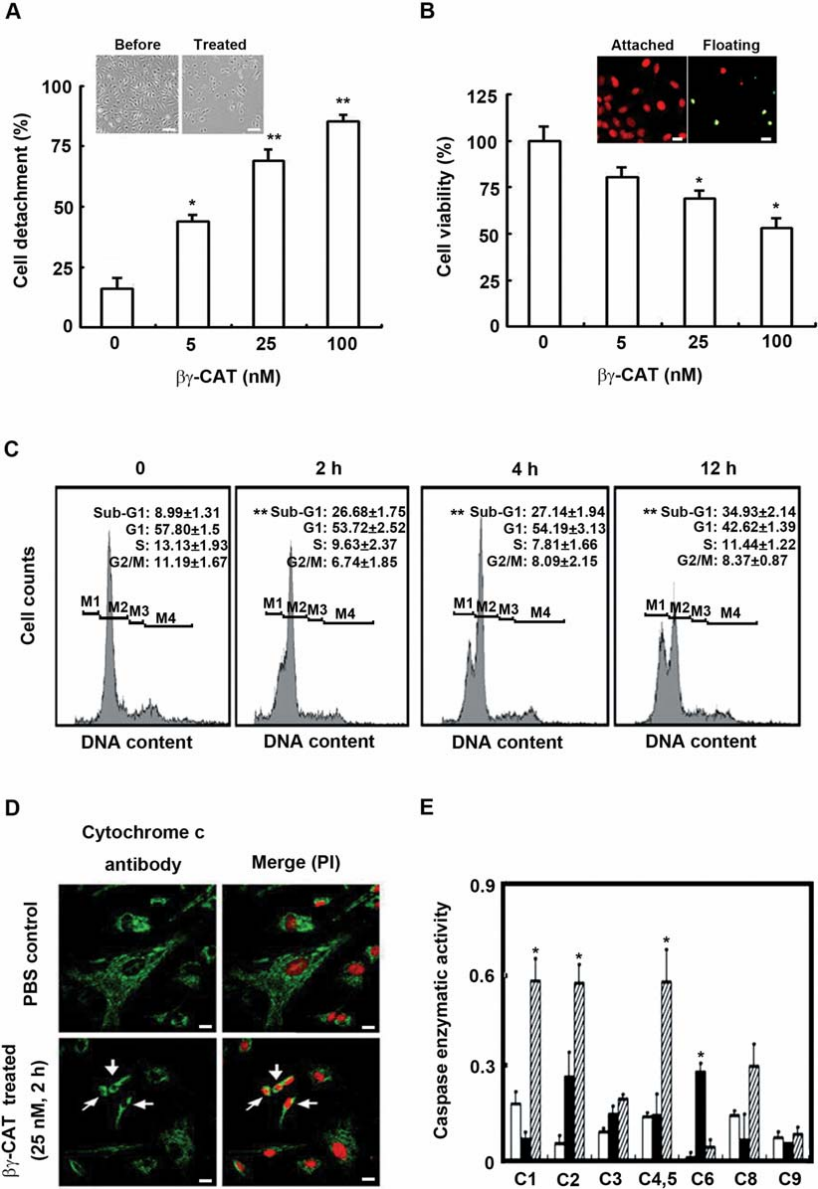
Induction of HUVEC detachment and apoptosis by high concentrations of βγ-CAT. (A) HUVEC detachment induced by various concentrations of βγ-CAT (treated for 2 h). Insert shows morphological changes of βγ-CAT treated HUVECs. Scale bars equal to 100 µm. (B) Cell viability of HUVECs after treated with βγ-CAT for 2 h as determined by MTT assay. Insert, TUNEL-staining of cells treated with βγ-CAT (25 nM, 2 h). Nucleus was counter-stained by PI (red channel). The merge (yellow) fluorescent signals indicate TUNEL positive staining. Scale bars equal to 20 µm. (C) The DNA content of HUVECs treated with βγ-CAT (25 nM) was detected using Hoechst by flow cytometry. Cells in the Sub-G_1_ (M1), G_1_ (M2), S (M3), and G_2_/M (M4) phases are indicated. (D) The release of cytochrome c was detected by indirect immunofluorescence. Nucleus was counter-stained by PI (red channel). Normal (top panels) and βγ-CAT treated cells (bottom panels) were labeled with a specific antibody against cytochrome c (green channel). Cells with cytochrome c releasing are marked by arrows. Scale bars equal to 20 µm. (E) Activated caspase activities of HUVECs after treated with βγ-CAT. White columns present PBS treated cells; black columns present the whole βγ-CAT treated cells; diagonal columns present the floating cells after βγ-CAT treated. In the parts of (D) and (E), HUVECs were treated by βγ-CAT (25 nM) for 2 h. Data were expressed as means±SEM of triplicate measurements (*, *p*<0.05; **, *p*<0.01, in A, B and E, compared with zero βγ-CAT; in C, compared with zero time point).

High concentrations of βγ-CAT caused cell detachment and interfered cell viability in a dose-dependent manner. After treatment of HUVECs with βγ-CAT (25 nM) for 2 h, 70% cells were detached (*p*<0.01, compared with zero βγ-CAT) (Fig. 7A), and βγ-CAT induced morphological changes of HUVECs as examined by a phase-contrast microscope (Fig. 7A insert). At the same time, the cell viability was decreased gradually as measured by MTT assay. Viable cells decreased to 69% of the control (*p*<0.05, compared with zero βγ-CAT) after treated with βγ-CAT (25 nM, 2 h) (Fig. 7B). Exposure of HUVECs to βγ-CAT (25 nM) resulted in time-dependent appearance of cells with a fragmented DNA content (sub-G_1_, *p*<0.01, compared with zero time point), as analyzed by flow cytometry (Fig. 7C). The apoptotic changes of HUVECs treated by βγ-CAT (25 nM) for 2 h were further determined by TUNEL staining, showing a substantial proportion of TUNEL-staining positive cells in floating fraction (Fig. 7B insert). In the same conditions, the release of cytochrome c from mitochondria of the cells treated was detected even in still attached cells (Fig. 7D). Furthermore, since apoptotic signals converge on the caspase cascade, the activities of caspase-1, -2, -3, -4/5, -6, -8 and -9 were investigated. Significant increase in the activities of caspase-1, -2, -4/5, and -6 were detected (*p*<0.05, compared with zero βγ-CAT) when tested with either floating fraction or the total cells (Fig. 7E).

### Cell vacuole formation and endocytosis of βγ-CAT

**Figure 8:**
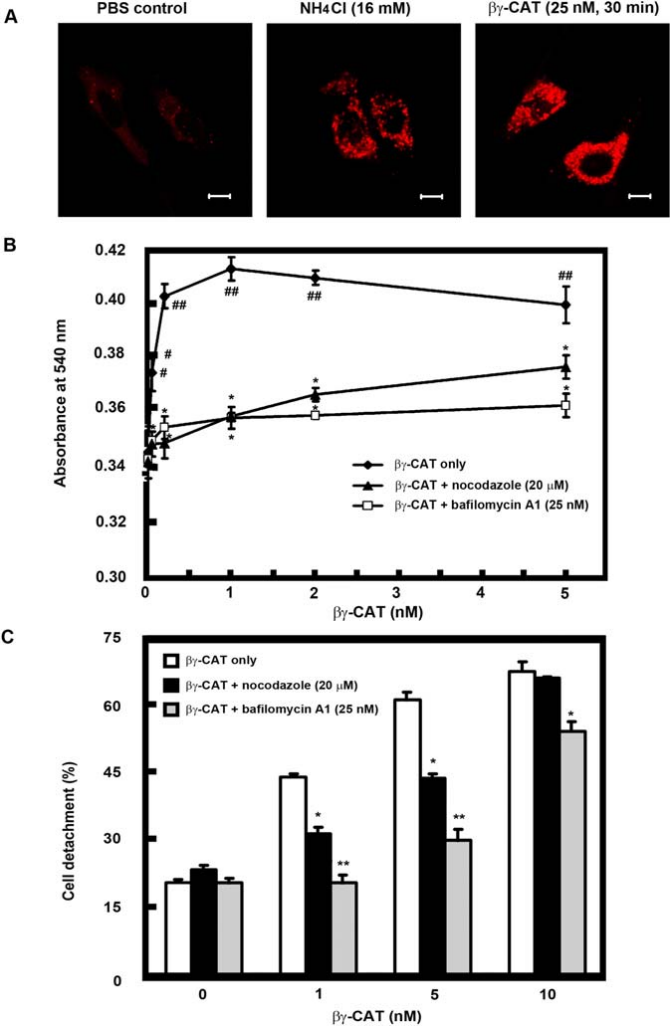
Vacuole formation of HUVECs induced by βγ-CAT and inhibition by bafilomycin A1 and nocodazole. (A) Extensive cell vacuolation induced by high dosages of βγ-CAT. HUVECs planted on glass coverslips were incubated with βγ-CAT (25 nM) for 30 min. PBS-treated cells were used as a negative control and cells treated with NH_4_Cl (16 mM) for 18 h were used as a positive control. The cells were stained with neutral red as described in “Methods”. The vacuole formation in the cells was observed by confocal microscope. Scale bars equal to 10 µm. (B) Neutral red uptake of βγ-CAT treated HUVECs in the absence and presence of bafilomycin A1 and nocodazole. HUVECs (2.5×10^4^ cells/ml) seeded in 96 well titer plates were incubated with bafilomycin A1 (25 nM) or nocodazole (20 µM) for 30 min at 37°C, then different concentrations of βγ-CAT (50 *p*M-5 nM) were added and incubated for another 30 min. Neutral red uptake was performed as described in “Methods”. (C) Inhibition of cell detachment induced by βγ-CAT in the presence of bafilomycin A1 and nocodazole. HUVECs planted in 24-well plates were incubated with bafilomycin A1 (25 nM) or nocodazole (20 µM) for 30 min at 37°C, then βγ-CAT (1 nM, 5 nM and 10 nM) was added and incubated for 5 h. Cell detachment was analyzed as described in “Methods”. Data were expressed as means±SEM of triplicate measurements (#, *p*<0.05; ##, *p*<0.01, compared with zero βγ-CAT (B); *, *p*<0.05; **, *p*<0.01, compared with the βγ-CAT only group (B–C).

Extensive and large intracellular vacuoles developed in HUVECs incubated with high dosages of βγ-CAT. Localization of neutral red, a membrane-permeant amine that rapidly localizes inside intracellular acidic compartments [25], within the vacuoles induced by βγ-CAT (25 nM, 30 min) was readily visible in 4 min after addition of the dye (Fig. 8A). Neutral red uptake, an objective and sensitive method for quantitative analysis of cell vacuole formation [25], was used to study cell vacuole formation induced by various concentrations of βγ-CAT. After low concentrations of βγ-CAT (50 *p*M-1 nM) treated, neutral red uptake by the cells was increased in a βγ-CAT dose-dependant manner, and reached to a plateau at 1 nM of the protein (*p*<0.01, compared with zero βγ-CAT) (Fig. 8B).

Bafilomycin A1 (25 nM), a specific inhibitor of the vacuolar-type ATPase [26]–[28], and nocodazole (20 µM), an agent of microtuble depolymerizing that is known to block trafficking between early and late endosomes [29], prevented the neutral red uptake of HUVECs induced by βγ-CAT (50 *p*M-5 nM, *p*<0.05, compared with the βγ-CAT only group), indicating that the vacuole formation was greatly inhibited (Fig. 8B). At the same time, in the presence of bafilomycin A1, the cell detachment induced by βγ-CAT significantly decreased (1 nM and 5 nM, *p*<0.01, 10 nM, *p*<0.05, compared with the βγ-CAT only group). The same inhibitory effect was also observed in cell detachment induced by βγ-CAT (1 nM and 5 nM, *p*<0.05) in the presence of nocodazole (20 µM) (Fig. 8C).

**Figure 9:**
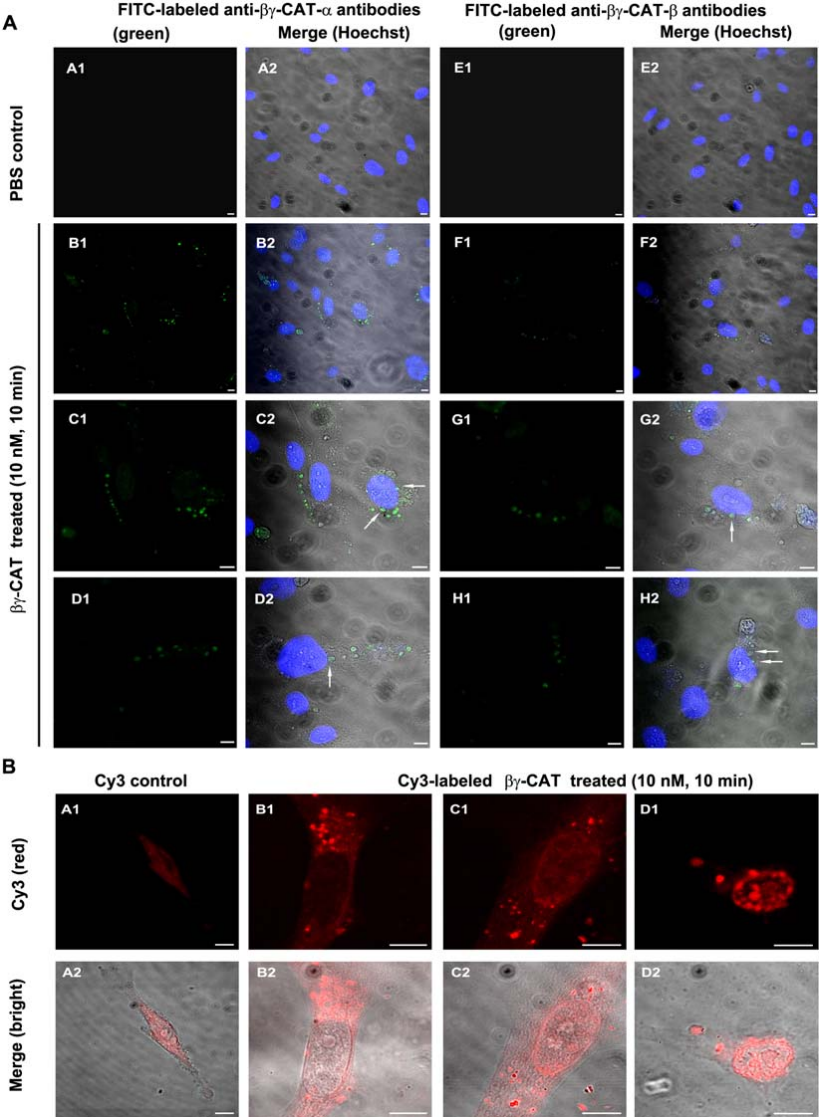
Endocytosis and intracellular localization of βγ-CAT. (A) The endocytosis and intracellular localization of βγ-CAT was detected by direct immunofluorescence. Nucleus was counter-stained by Hoechst (blue channel). PBS control (A1–A2, E1–E2) and βγ-CAT treated cells (10 nM, 10 min) were detected with FITC-labeled antibodies against the α-subunit (B1–D2) or β-subunit (F1–H2) (green channel). Some of the vacuoles were translocated to nucleus and partial fused with nuclear membrane, as indicated by arrows in C2, D2, G2 and H2. Scale bars equal to 10 µm. (B) Endocytosis of Cy3-labeled βγ-CAT. HUVECs were treated with Cy3-labeled βγ-CAT (10 nM, 10 min). The labeled protein (red channel) was observed to be concentrated in intracellular vacuoles (B1–D2). The vacuoles distributed in nuclear membrane were seen (D1–D2). Cells treated with free Cy3 dye (blocked with 20 mM Tris-HCl buffer, pH 7.8) was used as a negative control (A1–A2). Scale bars equal to 10 µm.

Fluorescein isothiocyante (FITC)-labeled rabbit polyclonal antibodies against each subunit of βγ-CAT were used to investigate the possible endocytosis and subcellular distribution of the protein by immunofluorescence. After addition of βγ-CAT (500 *p*M–25 nM) in cultured HUVECs, no cell surface binding of the protein could be observed. In contrast, βγ-CAT was observed to be rapidly internalized into the cells. As shown in Figure 9A, under a confocal microscope, the green-fluorescent signals of both subunits were mainly observed in intracellular vacuoles in HUVECs treated with 10 nM of βγ-CAT for 10 min. Some of the vacuoles were translocated to nucleus and partially fused with nuclear membrane, as indicated by arrows in Fig. 9A–C2, D2, G2 and H2. Alternatively, when HUVECs were treated with Cy3-labeled βγ-CAT (10 nM, 10 min), lots of red-fluorescent vacuoles were observed inside the cells and some of them were already concentrated in cell nucleus (Fig. 9B).

**Figure 10:**
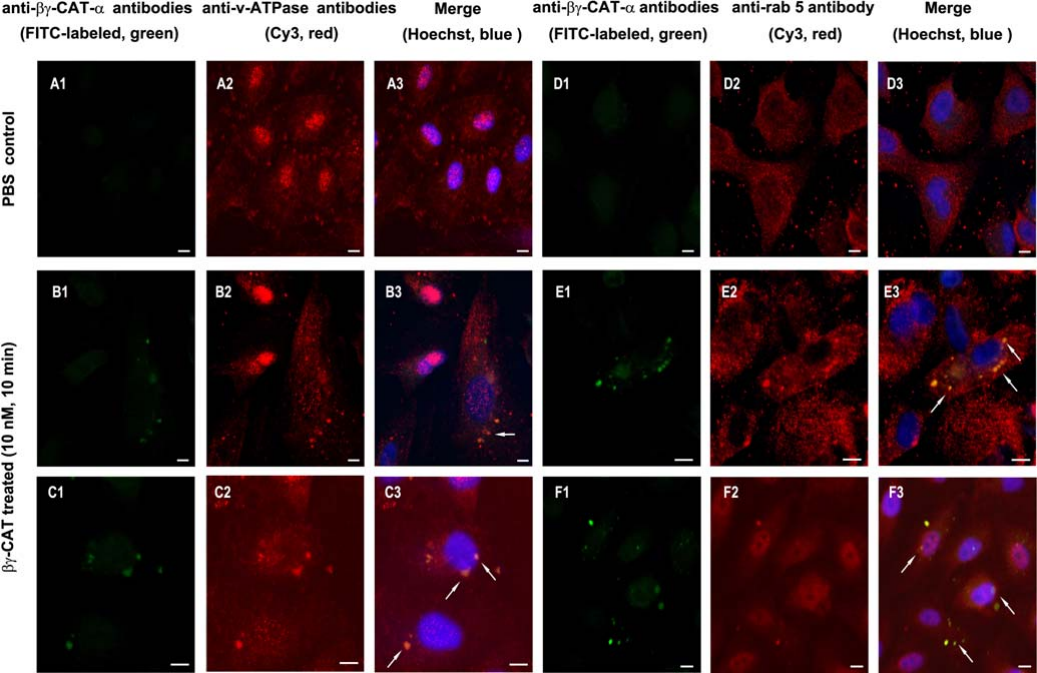
Co-localization of βγ-CAT positive vacuoles with endocytic marker proteins. PBS control (A1–A3, D1–D3) and βγ-CAT treated cells (10 nM, 10 min) (B1–C3, E1–F3) were first stained with specific antibodies against vacuolar type ATPase (A1–C3) and rab5 (D1–F3), respectively by indirect immunofluorescence (Cy3 labeled secondary antibodies, red channel). After washed with PBS (containing 1% BSA) for three times, the cells were stained again with FITC-labeled antibodies against βγ-CAT α-subunit (green channel). Nucleus was counter-stained by Hoechst (blue channel). Co-localization of βγ-CAT with vacuolar type ATPase (B3, C3) or rab5 (E3, F3) in intracellular vacuoles was indicated by arrows. The vacuoles reached to nuclear membrane were also observed, as indicated in C3, F3 by arrows. Scale bars equal to10 µm.

We next analyzed if, βγ-CAT positive vacuoles were endowed with endocytic proteins, like rab5, rab7 and vacuolar-type ATPase that are markers of different endocytic compartments involved in intracellular trafficking, with specific antibodies against rab5, rab7, vacuolar-type ATPase and βγ-CAT α-subunit. Among the endocytic proteins tested, rab5 and vacuolar-type ATPase, but not rab7, were found to be co-localized with βγ-CAT in some intracellular vacuoles, as indicated by arrows in Fig. 10-B3, C3, E3 and F3.

## Discussion

Over the past several decades, numerous studies have focused on the bioactive components existing in amphibian skin secretions. A lot of low molecular weight peptides with diverse biological activities have been identified and purified [30], [31]. These components can generally be classified as having either 1) regulatory or hormonal functions, or 2) antimicrobial activity. The peptides in the first group are analogues of mammalian hormones, neurotransmitters and growth factors. The antimicrobial peptides are an important part of the amphibian innate immune system. At present study, βγ-CAT, a protein with a native molecular weight of 72,000 Da that possesses potent hemolytic activity and lethal toxicity on mice has been purified from *B. maxima* skin secretions. In the final purification step, peaks II and III of Mono-Q column also showed hemolytic activity. There were three major proteins bands of 38-kDa, 22-kDa and 18-kDa in these fractions, and the protein bands of 38-kDa and 18-kDa could be recognized by antibodies against βγ-CAT in Western blotting analysis, suggesting the possible existence of βγ-CAT isoforms in the frog skin secretions. Similar to ep37 proteins, the precursor of βγ-CAT α-subunit has no signal peptide (see GenBank, EU003881). It might be released from cells through association with the β-subunit, a TFF with a signal peptide of 20 amino acids in its precursor (EU003882, Fig. 3C).

The β-subunit of βγ-CAT contains total 18 cysteine residues in the pattern of three repeats of consensus sequence C-X_9_-C-X_9_-C-X_4_-C-C-X_10_-C of TFFs [8], [9]. By homology, it could be predicted that they are paired in the same manner as that of mammalian TFFs. Unlike TFFs from mammals, the β-subunit does not possess additional cysteines, which are necessary in forming an extra disulfide bond in formation of homodimer and/or heterodimer in mammalian TFFs. Up to now, there are two forms of TFFs identified in *B. maxima* skin secretions. One is previously characterized single TFF (Bm-TFF2), which comprises two TFF domains [12]. Another one is the β-subunit of βγ-CAT at present study. In the purification process, Bm-TFF2 was completely separated from βγ-CAT. No association between Bm-TFF2 and the α-subunit of βγ-CAT was observed in the isolated fractions. In addition, similar to purified β-subunit of βγ-CAT, when tested with human and sheep erythrocytes, Bm-TFF2 did not show hemolytic activity even with a dosage used up to 1 µM.

Hemolytic effects of many protein toxins from animal, plant and microbial origins are often mediated by a specific receptor, which can be either a lipid membrane component, a membrane protein or carbohydrates [32]. In search for possible cell surface candidate receptor(s) of βγ-CAT, it was found that the hemolytic activity of βγ-CAT was not affected by pre-incubation of the protein with various lipid components and carbohydrates at 37°C for 30 min, including cholesterol, phosphatidylethanolamine, sphingomyelin, phosphatidylinositol, ceramide, phosphotidylserines, sphingosine, gangliosides, ω-Acetyl-D-Sphingosine, N-Acetyl-L-galactosamine, phosphatidylcholine, glucose, fucose, galactose, mannose, and lactose (Sigma, USA, concentrations used up to 10 mM). In addition, no binding of βγ-CAT to above lipids and carbohydrates was detected by enzyme-linked immunosorbent assays (ELISA). Furthermore, βγ-CAT did not show lysis effect on mimic liposomes that were made according to erythrocyte lipid compositions [33]. These results indicate that the receptor mediated βγ-CAT action might be a specific membrane protein, which may also explain the species specificity of hemolytic activity of βγ-CAT.

The biochemical characterization presented here reveals that βγ-CAT is able to form transmembrane pores in erythrocyte membranes. SDS-stable oligomers (>240-kDa) were detected by specific antibodies against βγ-CAT α-subunit (Fig. 5C). At present stage, it could not be excluded the possible contribution of some cell membrane proteins, like the membrane receptor(s) of βγ-CAT, in the formation of the oligomers. The α-subunit contains two βγ-crystallin domains in its N-terminal part. It has been reported that β-crystallins tend to make a transition from monomeric to oligomeric proteins [34], and protein S, a non-lens βγ-crystallin member from bacteria *Myxococcus xanthus* is able to oligomerize to form a multiplayer protective coat in stress conditions [35]. On the other hand, the main region of the C-terminal part of the α-subunit shows significant sequence homology to an internal fragment of ETX (a potent pore-forming protein) (Fig. 3B). This fragment contains domain II of the toxin, which is thought to be important in the insertion of the toxin into the membrane [36]. Thus, the α-subunit possesses structural features for membrane pore formation via oligomerization.

In mammals, one of the fundamental actions of TFFs is to promote epithelial-cell restitution, a process that promotes epithelial-cell migration to reseal superficial wounds after injury, as demonstrated in gastrointestinal mucosal healing [13], [14]. The finding that βγ-CAT could also induce HUVEC migration and show wound healing effects is consistent with the functions of mammalian TFFs. However, the concentrations (25–50 *p*M) used for the determined activity of βγ-CAT were extremely low, compared with micromolar concentrations of single mammalian TFF peptides used in similar assays [37]. At high concentrations (5–100 nM), βγ-CAT caused HUVEC detachment and induced cell apoptosis. The release of cytochrome c, an initiator of intrinsic apoptotic pathway, from mitochondria of the cells treated was detected even in still attached cells (Fig. 7D), indicating that the apoptotic changes were initiated before the cell detachment. Thus, βγ-CAT appears to be able to modulate different aspects of cell physiology, and is not simply a “cytotoxic protein”.

βγ-CAT was able to induce multiple cellular responses in HUVECs. Microarray analysis of the gene expression profile of the cells treated with βγ-CAT (25 nM, 2 h) (Supporting information Materials and Methods S1) showed that the expression of 123 genes was significantly changed (fold change≥3, *q* value = 0, FDR (false discovery rate) = 0). 121 genes were up-regulated, among which members of nuclear receptor subfamily 4 (NR4A) were the most prominent. Only 2 genes were down-regulated (including collagen type I) (Supporting information Fig. S1 and Table S1). Members of NR4A exhibit differential roles in determining cell growth or cell death depending on its location in the nucleus or mitochondria. NR4A1 (also called Nur77, TR3) mediates cell apoptosis through translocation from nucleus to mitochondria and induction of cytochrome c releasing [38]. Significant activation of NR4A members suggests these orphan nuclear receptors may involve in the mediation of βγ-CAT biological functions, like proapoptotic action of the protein at high concentrations. Up-regulated expression of a number of matrix metalloproteinases by βγ-CAT could facilitate cell migration and detachment [39]. βγ-CAT also induced the expression of a number of cytokines, including interleukin 1α, interleukin 8 and interleukin 6, as well as various chemokines in HUVECs. These data provide clues for study in detail of molecular pathways through which βγ-CAT exerts its biological functions.

Neutral red staining and uptake assays provide evidences that βγ-CAT is able to induce cell vacuole formation at different concentrations (from *p*M to nM). Diminished neutral red uptake with higher dosages of βγ-CAT was due to cell detachment. Existence of βγ-CAT in intracellular vacuoles and co-localization with endocytic marker proteins, such as rab5 and vacuolar type ATPase, indicate that βγ-CAT positive vacuoles originated from endocytic route. Furthermore, translocation of βγ-CAT positive vacuoles to cell nucleus and even partial fusion with nuclear membrane were observed (Figs. 9 and 10, marked by arrows). In addition, bafilomycin A1 and nocodazole, which inhibited βγ-CAT induced vacuole formation and endocytosis, could also inhibit its cellular functions, as shown by significantly inhibited cell detachment induced by βγ-CAT (Fig. 8B and 8C). These results suggest that vacuole formation and endocytosis of βγ-CAT play an important role in its cellular functions. Figure 8C also revealed that the inhibitors, with the dosages used (bafilomycin A1 25 nM, nocodazole 20 µM), were not able to inhibit the cell detachment induced by high concentrations of βγ-CAT (>10 nM). Higher dosages (bafilomycin A1>50 nM, and nocodazole>40 µM) or long time treatment (over 24 h) of the inhibitors in cultured cells could influence cell viability and mobility, and effort to test the inhibitory effects of bafilomycin A1 and nocodazole on βγ-CAT induced cell apoptosis and cell migration was unsuccessful. Extensive cellular vacuolation was observed when HUVECs were treated with a high dosage of βγ-CAT (25 nM) (Fig. 8A). This might be one of reasons to explain the cell apoptosis induced by βγ-CAT at high concentrations, as in the case of bacterial *Helicobacter pylori* vacuolating toxin VacA that causes cellular swelling, expansion of endosomal compartments, extensive vacuolation and finally cell death [40].

In this line, βγ-CAT is similar to a number of pore-forming protein toxins of bacterial and plant origins that have cytosolic targets. These protein toxins must be endocytosed and translocated into the cytosol where they exert their cytotoxic actions [41]. For example, the bacterial adenylate cyclase toxin and Shiga toxin, with hemolytic activity on human erythrocytes *in vitro*, bind their respective receptors at the cell surface, and then negotiating intracellular trafficking pathways that the cell maintains for endogenous protein transport, traffic from the cell surface to the endoplasmic reticulum [42], [43].

It seems that distinct molecular forms of frog skin TFFs may exert their functions in different models. One is that Bm-TFF2 (single TFF) binds to human platelet membrane in a saturable manner and triggers down-stream signaling cascades, leading to platelet activation [12]. Another one is the case of βγ-CAT, in which a TFF (the β-subunit) combines with a non-lens βγ-crystallin member (the α-subunit). The resulted complex is rapidly endocytosed and translocated to cytosolic targets. Nucleus is a potential candidate for βγ-CAT, as shown in Figures 9 and 10, suggesting that endocytosis, nuclear translocation and possible transcriptional regulation directly or indirectly might be one potential action ways of βγ-CAT. Since in mammals, the mechanisms by which TFFs exert their biological activity are still largely unknown. So far, no “receptor” that can mediate the functional effects of TFFs has yet been identified, leading to the speculation that they might not act alone [14]. It is highly interesting to investigate whether the similar action mechanisms might exist in mammals.

With rabbit polyclonal antibodies against each subunit of βγ-CAT, distribution of βγ-CAT and the homologues of its each subunit in frog skin was observed around the epidermis and granular glands imbedded in the dermis (Supporting information Fig. S2). In addition, immuno-reactivity was also determined in the intestine and stomach of the frog, indicating that βγ-CAT and/or the homologues of its each subunit are expressed in these tissues. This result is similar to observed tissue distribution of ep37 proteins in newt *C. pyrrhogaster* [2], [3], AIM1 gene family products in mammals [5], [6], and TFFs in amphibian and mammals [10], [11], [22]. Frog skin is naked and constantly confronted by a complex mixture of potentially injurious mechanical and chemical factors. Constant skin renewal and repair in disruptions of the surface layer of cells occur frequently. Our study has revealed the presence of a single TFF (Bm-TFF2) [12] and a complex of a non-lens βγ-crystallin and a TFF (βγ-CAT, present study) in *B. maxima* skin. It is reasonable to hypothesize that rich distribution of such proteins in frog skin, especially in epidermis, could play important physiological roles in skin tissue repair and maintaining of tissue homeostasis. Alternatively, by possessing potent toxicity on mammals, βγ-CAT may function directly or indirectly as a defensive substance against predators.

In conclusion, a naturally existing non-lens βγ-crystallin and trefoil factor complex, βγ-CAT, has been isolated and characterized for the first time from amphibian skin secretions. βγ-CAT is highly active and shows multiple effects on mammalian cells, including hemolysis via membrane pore formation, induction of cell migration, cell detachment and apoptosis depend on the dosages used. Present study also revealed the endocytosis and translocation of βγ-CAT to cytosol targets (most probably nucleus), which may play important role in its cellular functions. These findings illustrate novel cellular functions of non-lens βγ-cyrstallin members and action mechanism via association with TFFs, serving as clues for investigating the possible occurrence of similar molecules and action mechanisms in mammals. TFFs have been developed as clinic drugs in treatment of gastrointestinal mucosal healing. Due to its strong and multiple pharmacological activities, βγ-CAT might be a useful model molecule for design and generation of new drugs for wound healing and other human diseases.

## Materials and methods

### Ethics

All the animal studies were reviewed and approved by the animal care and use committee of Kunming Institute of Zoology, The Chinese Academy of Sciences. For the usage of human cells, the study was approved by local review boards for ethics in Kunming Blood Center and The First Affiliated Hospital of Kunming Medical College, and written consent was obtained from healthy subjects. We took blood or umbilical vein samples from the healthy subjects after obtaining informed consent.

### Electrophoresis

SDS-PAGE and native PAGE were performed according to Laemmli [44]. For SDS-PAGE, samples were pretreated in 2.5% SDS alone (non-reducing conditions) or in 2.5% SDS and 5% β-mercaptoethanol (reducing conditions) at 100°C for 6 min. Electrophoresis was carried out at 5 V/cm for 1.5 h. Protein bands were stained with silver (AgNO_3_ technique) [45] or with Coomassie Brilliant Blue R-250. For the quantitative analysis of the molecular weight and their relative percentage of protein bands, the gel was stained with 0.1% Coomassie Brilliant Blue R-250, scanned by HP Scan Jet 4200C scanner, and then analyzed by Gel-Pro analyzer (version 3.1) software.

### Gel filtration analysis of native molecular weight of βγ-CAT

The native molecular weight of βγ-CAT was analyzed by gel filtration as described [46]. Briefly, purified βγ-CAT (1.0 mg) was loaded on a Sephadex G-100 column (superfine, 2.6×100 cm). The elution was performed with 50 mM Tris-HCl, pH 7.5, containing 100 mM KCl at a flow rate of 0.2 ml/min. The following proteins were used as standards: thyroglobulin (305-kDa), phosphatase B (97-kDa), bovine serum albumin (66-kDa), Ovalbumin (45-kDa), Chymotrypsin (25-kDa) (Sigma, USA).

### Sequence analysis

Purified βγ-CAT was run on a 10% SDS-PAGE gel under reducing conditions and then transferred to a PVDF membrane (Millipore product, USA). After briefly stained with Coomassie brilliant R-250, protein bands corresponding to each subunit were cut out and the N-terminal sequence was determined by Edman degradation on an ABI model 476A protein sequencer. For internal amino acid sequences of the β-subunit, HPLC C_4_ column purified β-subunit was digested by trypsin, and the resultant fragments were separated by HPLC on a C_8_ column. To obtain the internal amino acid sequences of the α-subunit, purified βγ-CAT was digested by trypsin, the resultant fragments were separated by successive HPLC purification first on a C_4_ column, then on a C_8_ column. Purified peptides were analyzed with the protein sequencer.

### Molecular cloning of βγ-CAT

For cloning of the β-subunit, a cDNA internal fragment of the β-subunit was first obtained with PCR amplification by using degenerated oligonucleotide primers designed according to determined N-terminal and internal peptide sequences of the subunit. Briefly, total RNA was prepared from the frog skin by an RNA extract kit (Invitrogen). Single-stranded cDNAs were prepared from mRNAs contained in the total RNA (5 µg) by reverse transcriptase (Invitrogen), using an oligo-d(T)_18_ primer. PCR products were subcloned into a pGEM-T vector (Promega). From the cDNA fragment sequence of the β-subunit obtained from the first PCR amplification, the specific primers of the subunit were designed and used in a PCR-based method for high stringency screening of a full-length β-subunit encoding clone from a *B. maxima* skin cDNA library as described [18].

Since at the beginning, the arrangement of obtained βγ-CAT α-subunit internal sequences in the mature polypeptide was unknown, a group of degenerated oligonucleotide primers both in sense and antisense directions corresponding to the internal α-subunit sequences were designed. Totally, 15 sense and 17 antisense primers were synthesized. They were used in different combinations by paring each sense and antisense primers in PCR amplifications. From a cDNA fragment sequence of the α-subunit obtained from the first PCR amplifications, specific primers were designed and used in screening of a full-length α-subunit encoding clone as mentioned above. The deduced protein sequences were also confirmed by subjecting isolated α- and β-subunits to peptide mass fingerprinting analysis. The molecular masses of the peptides digested by trypsin were estimated by a Voyage-DE PRO instrument (Applied Biosystems, USA).

### Preparation of rabbit polyclonal antibodies against native βγ-CAT and each subunit

Due to the hydrophobic properties of βγ-CAT α-subunit, its recovery rate was very low by HPLC isolation. Alternatively, purified βγ-CAT was run on a 10% SDS-PAGE gel under reducing conditions, and the protein bands corresponding to the α- and β-subunits on the gel were extracted by 0.01M NH_4_HCO_3_ with 0.05% SDS. The purity of each subunit recovered from the gel was tested by SDS-PAGE. Protein concentration was determined by a protein assay kit (Bio-Rad, CA, USA) with BSA as a standard. Each subunit of βγ-CAT recovered from SDS-PAGE gel and native βγ-CAT were used as antigens to immunize male New Zealand white rabbits in our laboratory. The polyclonal antibodies (IgG) were purified from the harvested rabbits sera by double ammonium sulfate precipitations (35% final concentration), and then dialyzed against PBS. The specificity of the antibodies against each subunit was confirmed by ELISA, and no cross-reactions between them were detected. The negative control IgG was purified from pre-immunized rabbit sera.

### Measurement of hemolysis

Erythrocytes from various species were washed three times with PBS buffer (137 mM NaCl, 1.5 mM KH_2_PO_4_, 2.7 mM KCl, 8.1 mM Na_2_HPO_4_) and then re-suspended in the same buffer. Erythrocytes (5×10^6^ cells/ml) were incubated with various concentrations of βγ-CAT (15 *p*M–30 nM) at 37°C for 30 min, and centrifuged at 500× *g* for 5 min at 4°C. The supernatant obtained was assayed for absorbance at 415 nm. 100% lysis was defined as the absorbance of the supernatant obtained from 0.1% Triton X-100 lysed samples. To investigate the neutralizing capacity of the antibodies against each subunit of βγ-CAT on its hemolytic activity, βγ-CAT (3 nM) was incubated with a series diluted rabbit specific antiserum against each subunit at 37°C for 30 min, then the rest of βγ-CAT hemolytic activity on human erythrocytes was determined as above.

### Intracellular K^+^ efflux

Human erythrocytes were washed 3 times (centrifuged at 4°C, 2000× *g*, 5 min) in Hank's balanced salt solution, pH 7.4 (HBSS, MgSO_4_ 0.6 mM, KCl 5.4 mM, CaCl_2_ 1.3 mM, MgCl_2_ 0.5 mM, Glucose 5.6 mM, NaH_2_PO_4_ 0.4 mM, NaHCO_3_ 4.2 mM, NaCl 137 mM, Na_2_HPO_4_ 0.3 mM). Washed erythrocytes (4×10^9^ cells/ml) were incubated with various concentrations of βγ-CAT (0.15–150 nM) at 37°C for 30 min, then centrifuged. Aliquots of the supernatant were taken to measure hemolytic rate as mentioned above. The rest of the supernatant was used to detect K^+^ concentration by an Instrumentation Laboratory model 6400A flame photometer. The total K^+^ concentration was determined by cells lysed with 1% Triton X-100.

### Functional diameter of membrane pores

The functional size of βγ-CAT formed membrane pores was determined as described [47]. Briefly, PEGs (Fluka) with various molecular sizes were added in PBS with a concentration equivalent to 40 mosM to counterbalance the osmotic pressure of intracellular hemoglobins [48], and total osmotic pressure of the solutions was adjusted to 295 mosM. Washed human erythrocytes were suspended in PEG solutions (4×10^9^ cells/ml), and the hemolytic activity of βγ-CAT was measured as above. The hydrodynamic diameters of PEGs are from the reports [24], [47].

### Western blotting

Human erythrocytes (6×10^7^ cells/ml) were incubated with various concentrations of βγ-CAT (0.5–3 nM) in 1 ml of PBS at 37°C for 30 min. The erythrocytes were collected by centrifugation at 22,000× *g* for 10 min at 4°C and washed three times with 5 mM Tris-HCl buffer (pH 7.2). The erythrocyte membranes were incubated with 2% (w/v) SDS at 37°C for 30 min, loaded on a SDS-PAGE gel (linear gradient acrylamide gel of 3–15%) and then electrotransferred to a PVDF membrane (Millipore product, USA) as previously described [49]. The rabbit polyclonal antibodies against native βγ-CAT, the α- and β-subunits (500-fold diluted) were used to detect βγ-CAT subunits followed by peroxidase-coupled goat anti-rabbit antibodies (Santa Cruz, CA). Bound antibody was detected using chemiluminescence (SuperSignal West Pico Chemiluminescent Substrate, PIERCE, USA).

### Cell culture

Primary cultured HUVECs were obtained by established method [50]. The identity of the cells was confirmed by positive staining of indirect immunofluorescence with a monoclonal antibody of thrombomodulin (Abcam, MA). Cells used in the experiments were confluent monolayers between passages 2 and 3.

### Cell migration and wound healing

HUVEC migration was tested by using a modified Boyden chamber assay according to the instructions of the manufacturer (Chemicon, CA). Briefly, 5×10^6^ cells were planted into each chamber (8 µm pore, collagen type I pre-coated). Different concentrations of βγ-CAT (25–50 *p*M) and vascular endothelial growth factor (VEGF, 50 *p*M) (Sigma, USA) were added into the upper of chambers for 24 h. Migrated cells were dyed with crystal violet. Photography was recorded by an inverted-phase microscope. The migration activity was expressed as the value monitored at 550 nm of extraction.

Wound healing assay was performed mainly as reported [51]. HUVECs in six-well collagen type I pre-coated culture plates (Nunc, USA) were scratched by a standard 1-ml pipette tip across the diameter of the wells. The medium and non-adherent cells were aspirated off, and the plates were rinsed twice with starve medium (Medium 199, Gibco, USA) containing 2% fetal bovine serum (Hyclone, USA) only. Fresh starve medium containing βγ-CAT (25–50 *p*M) or VEGF (50 *p*M) was added into the plates and changed every 24 h. Time-lapse photography of the wounding edges was performed under an inverted-phase microscope within 72 h.

### Cell detachment and cell viability

HUVECs (cultured in 35 mm dishes) were treated with βγ-CAT (5–100 nM) for 2 h at 37°C in a 5% CO_2_ incubator. The floating cells were pooled. Photomicrography was taken by an inverted-phase microscope. The remaining monolayer was detached by trypsin (0.25% in PBS, containing 0.02% EDTA) for 5 min. The floating and attached cells were stained with 0.4% Trypan Blue (Sigma, USA) and counted, respectively. Percentage of the detachment was expressed as (total cells in the supernatant and wash)/(total cells in supernatant, wash, and detached by trypsin) × 100%. In parallel experiments, the cell viability was detected by a MTT method [52].

### Apoptosis assays

For DNA content measurement and cell cycle analysis, HUVECs (cultured in 25 cm^2^ flask) were incubated with βγ-CAT (25 nM) for different times. Total cells were collected, and fixed with 70% ice-cold ethanol. DNA was dyed by Hoechst 33342 (Sigma, USA) and analyzed by flow cytometry (FACSVantage SE, Becton Dickinson, NJ, USA) [53]. Data were collected and analyzed from a minimum of 10,000 events per sample.

DNA fragmentation of the cells was assayed by a TUNEL-staining kit (Molecular Probe, OR). The cytochrome c release of the cells was detected with a cytochrome c assay kit (Molecular Probe, OR). Briefly, HUVECs were cultured on glass coverslips pre-coated with collagen (Sigma, USA) and treated by βγ-CAT (25 nM) for 2 h. At the end of incubation, cytochrome c was detected by a specific antibody following the instructions of the manufacturer. Nucleus was counter-stained by propidium iodide (PI). The coverslips were observed under a confocal microscope (LSM510 META, Zeiss). Caspases activities of HUVECs (cultured in 25 cm^2^ flask) were assessed with a caspase substrate set IV kit (Calbiochem, USA), using 7-amino-4 (trifluoromethyl) coumarin (AFC) fluorogenic as a substrate. The activities were expressed as the pure maximal increased fluorescence per hour per µg of total proteins [54].

### Measurement of cell vacuole formation and inhibition by bafilomycin A1 and nocodazole

Cell vacuole formation induced by βγ-CAT was assessed by neutral red uptake assay as described [25]. HUVECs planted on glass coverslips were treated with βγ-CAT (25 nM) for 30 min at 37°C. The cells were washed with PBS and then stained with 0.05% neutral red (Amersco, USA) for 4 min at 37°C. After washed with PBS again, the cells were observed immediately under a confocal microscope at 645 nm by 488 nm excitation. PBS-treated cells were used as a negative control and cells treated with NH_4_Cl (16 mM) for 18 h were used as a positive control. For quantitative neutral red uptake assay, HUVECs (2.5×10^4^ cells/ml) seeded in 96 well titer plates were incubated with different concentrations of βγ-CAT (50 *p*M-5 nM) for 30 min at 37°C. Afterwards, the cells were washed with PBS and stained with 0.05% neutral red for 4 min at 37°C. The neutral red taken by the cells was extracted by the addition of acidified ethanol, and then the optical density (OD) of the extracted solution was determined by a spectrophotometer at 540 nm [25].

To test the inhibitory effects of bafilomycin A1 and nocodazole (Sigma, USA) on cell vacuole formation induced by βγ-CAT, HUVECs in 96-well titer plates were incubated with bafilomycin A1 (25 nM) or nocodazole (20 µM) for 30 min at 37°C. Then βγ-CAT (0–5 nM) was added and the cells were incubated for 30 min at 37°C. Cell vacuole formation was determined by the neutral red uptake assay as described above. For testing the inhibitory activity of bafilomycin A1 and nocodazole on cellular functions of βγ-CAT, HUVECs in 24-well plates were incubated with bafilomycin A1 (25 nM) or nocodazole (20 µM) for 30 min at 37°C. Then the cells were incubated with various dosages of βγ-CAT (1–10 nM) for 5 h at 37°C. Cell detachment was assayed as mentioned above.

### Endocytosis of βγ-CAT and co-localization with endocytic marker proteins

To investigate the endocytosis and subcellular distribution of βγ-CAT in HUVECs, βγ-CAT (10 nM) was incubated with HUVECs cultured on glass coverslips for 10 min at 37°C. The cells were washed three times with ice-cold PBS, and fixed with 4% paraformaldehyde for 30 min. Afterwards, the cells were permeabilized by 0.01% NP40 for 20 min at room temperature and blocked with a solution of PBS (containing 1% BSA). The cells were subsequently incubated with FITC-labeled rabbit polyclonal antibodies against each subunit of βγ-CAT, respectively. After washed three times by PBS (containing 1% BSA) to get rid of non-specific binding, nucleus was counter-stained by Hoechst. PBS treated cells were used as a negative control. The coverslips were viewed under the confocal microscope with a Plan-Neofluar 40×1.3 oil objective. Images were acquired using a confocal system (LSM 510 Image Examiner Installation, Zeiss). Alternatively, βγ-CAT was directly labeled with Cy3 dye according to the manufacture's instructions (Amersham Biosciences, USA). The Cy3-labeled protein was separated from free Cy3 dye by a Sephadex G-25 column. Labeled βγ-CAT (10 nM) was incubated with HUVECs cultured on glass coverslips for 10 min at 37°C. The cells were washed three times with PBS, fixed with 75% ethanol, and then observed under the confocal microscope with a Plan-Apochromat 100×1.4 oil objective. Free Cy3 dye (pre-blocked with 20 mM Tris-HCl, pH 7.8) was used as a negative control.

For analysis of intracellular co-localization of βγ-CAT with endocytic marker proteins, HUVECs seeded on coverslips were incubated with βγ-CAT (10 nM) at 37°C for 10 min, and then fixed, permeabilized and blocked as described above. The cells were incubated with rabbit polyclonal antibodies against vacuolar type ATPase A1 (Santa Cruz Biotechnology, USA), a monoclonal antibody against rab5 (R7904, Sigma, USA) or a monoclonal antibody against rab7 (R8779, Sigma, USA), and then stained with Cy3-labeled secondary antibodies. After washed three times with PBS (containing 1% BSA), the cells were incubated with FITC-labeled rabbit polyclonal antibodies against βγ-CAT α-subunit for 1 h at 37°C. Nucleus was counter-stained by Hoechst. The coverslips were observed under the confocal microscope with a Plan-Neofluar 40×1.3 oil DIC objective. Images were acquired using the confocal system.

### Statistical analysis

Data were analyzed with student's t-test for variance. Experimental values expressed as means±SEM. The level of statistical significance was set at the level of *p*<0.05.

### Data deposition

The nucleotide sequence data reported in this paper are available from GenBank database with accession numbers of EU003881 (βγ-CAT α-subunit) and EU003882 (βγ-CAT β-subunit). Gene expression data with Affymetrix chips were deposited in the National Center for Biotechnology Information Gene Expression Omnibus database (NCBI GEO accession number of GSE10479).

## Supporting Information

Materials and Methods S1 (0.05 MB DOC)

Figure S1 Hierarchical clustering of all significantly differential expression genes of HUVECs induced by betagamma-CAT. Four independent biological replicates of HUVECs treated with betagamma-CAT (25 nM, 2 h) were compared with normal control by SAM. The significantly differential expression of 123 genes (fold change≥3, q value = 0, FDR (false discovery rate) = 0) are shown (columns show treated cells organized anatomically and rows present genes organized by hierarchical clustering). A color tag represents expression levels, with red representing the highest levels and green representing the lowest levels of expression.
(1.74 MB TIF)

Figure S2 Distribution of betagamma-CAT and/or the homologues of its each subunit in frog B. maxima skin, intestine and stomach. Frog tissue cryostat sections were prepared as described in supporting information. Serial sections were stained with rabbit polyclonal antibodies against betagamma-CAT alpha-subunit (D1–F2), beta-subunit (G1–I2), respectively (green channel). Pre-immunized rabbit IgG (A1–C2) was used as control. Nucleus was counter-stained with PI (red channel). The same cryostat sections were observed by phase contrast in order to demonstrate morphology (A1, B1 and C1; D1, E1 and F1; G1, H1 and I1). Scale bars equal to 100 µm. In A2, D2 and G2, S, serous glands; L, lipid glands; M, mucous glands; D, dermis; E, epidermis.
(6.41 MB TIF)

Table S1 All significantly differential expression of 123 genes of HUVECs induced by betagamma-CAT, as listed in Excel file.
(0.03 MB XLS)
