# Supplementary material for: A Novel Non-Lens βγ−Crystallin and Trefoil Factor Complex from Amphibian Skin and Its Functional Implications
Source: PLoS One. 2008 Mar 12;3(3):e1770. doi: 10.1371/journal.pone.0001770 (PMC2262142; doi:10.1371/journal.pone.0001770)
Supplement: Materials and Methods S1 — (0.05 MB DOC) [file pone.0001770.s001.doc]

**Supporting Information**

**Materials and methods**

**Microarrays and data analysis**

Affymetrix human genome-U133 plus 2.0 chips (47,000 transcripts, Affymetrix, CA, USA) were used to evaluate genome wide gene expression levels. Total RNA was extracted with Trizol reagent (Invitrogen, CA, USA) from 4 independent biological replicates of primary cultured HUVECs treated with βγ-CAT (25 nM, 2 h) and PBS-treated cells as a normal control. The microarray experiments were performed at CapitalBio Corporation (Beijing, China) whose GeneChip microarray service was certificated by Affymetrix. Global median normalization by dChip and flagged spots corresponding to absent or low-quality signals (<100) were removed before the analysis. SAM (Significance analysis of microarrays) software was used to find differentially expressed genes (fold change≥3, *q* value =0, FDR (false discovery rate) =0) (Tusher VG, Tibshirani R, Chu G (2001) Proc Natl Acad Sci U S A 98: 5116-5121). To focus analysis on the greatest variation genes in expression, we selected genes whose expression changed above 3-fold from their average expression across the entire set of the samples. We then performed uncentered hierarchical clustering (Eisen MB, Spellman PT, Brown PO, Botstein D (1998) Proc Natl Acad Sci U S A 95: 14863-14868) by using average-linkage clustering to group the genes based on similarity in expression patterns.

**Immunohistochemistry**

Frogs were cooled for 15 min at 4 °C to anesthetize the animals. Snap frozen fresh tissues of the frog in liquid nitrogen and embedded with Tissue-Tek O.C.T. (Sakura Finetek, USA). Cut 10 *μ*m cryostat cross-sections and mount on poly-L-lysine coated slides, storing the slides at –80 °C until needed. Before immunofluorescence staining, the sections were rapidly warmed up at 37 °C for 5 min, and then fixed with pre-cooled (–20 °C for 30 min) acetone for 10 min at room temperature. Rinsed the sections three times with ice cold PBS. The sections were blocked with PBS (containing 5% BSA) for 1 h at room temperature, and then incubated for 1 h at room temperature with rabbit polyclonal antibodies against each subunit of βγ-CAT  (1:100 dilution), respectively. The pre-immunized rabbit IgG was used as a negative control. The FITC-labeled goat anti-rabbit antibodies (Santa Cruz, CA, USA) was employed as secondary antibodies and incubated with the sections for 1 h. After washed three times with PBS, nucleus was counter-stained with PI. The sections were observed under a confocal microscope (LSM 510 Image Examiner Installation, Zeiss, Plan-Neofluar 20×/0.5). Images were captured using a confocal system (LSM 510 Image Examiner Installation, Zeiss).
