# Supplementary figures and images for: A Novel Non-Lens βγ−Crystallin and Trefoil Factor Complex from Amphibian Skin and Its Functional Implications
Source: PLoS One. 2008 Mar 12;3(3):e1770. doi: 10.1371/journal.pone.0001770 (PMC2262142; doi:10.1371/journal.pone.0001770)

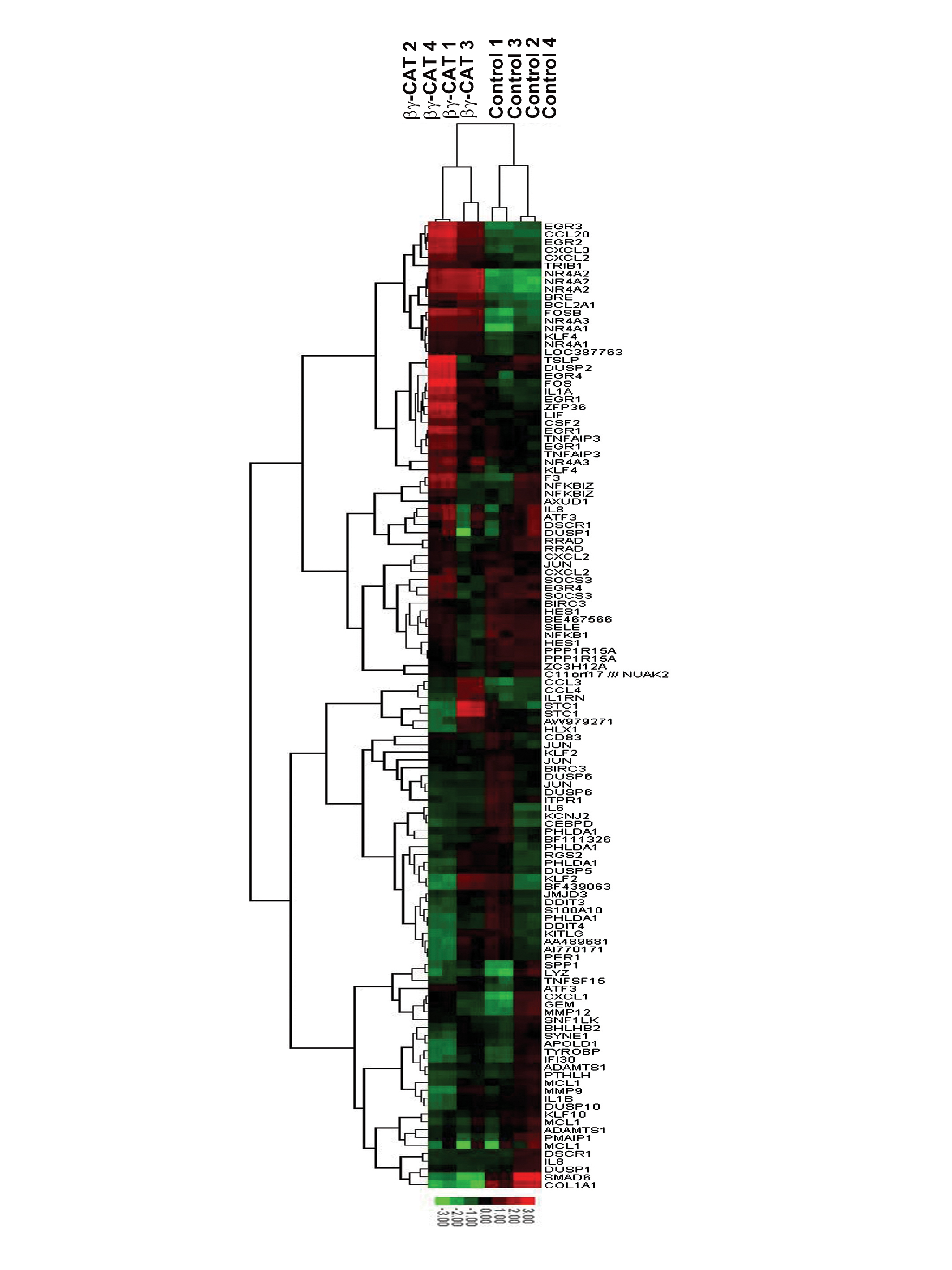

Supplement: Figure S1 — Hierarchical clustering of all significantly differential expression genes of HUVECs induced by betagamma-CAT. Four independent biological replicates of HUVECs treated with betagamma-CAT (25 nM, 2 h) were compared with normal control by SAM. The significantly differential expression of 123 genes (fold change≥3, q value = 0, FDR (false discovery rate) = 0) are shown (columns show treated cells organized anatomically and rows present genes organized by hierarchical clustering). A color tag represents expression levels, with red representing the highest levels and green representing the lowest levels of expression. (1.74 MB TIF) [file pone.0001770.s002.tif]

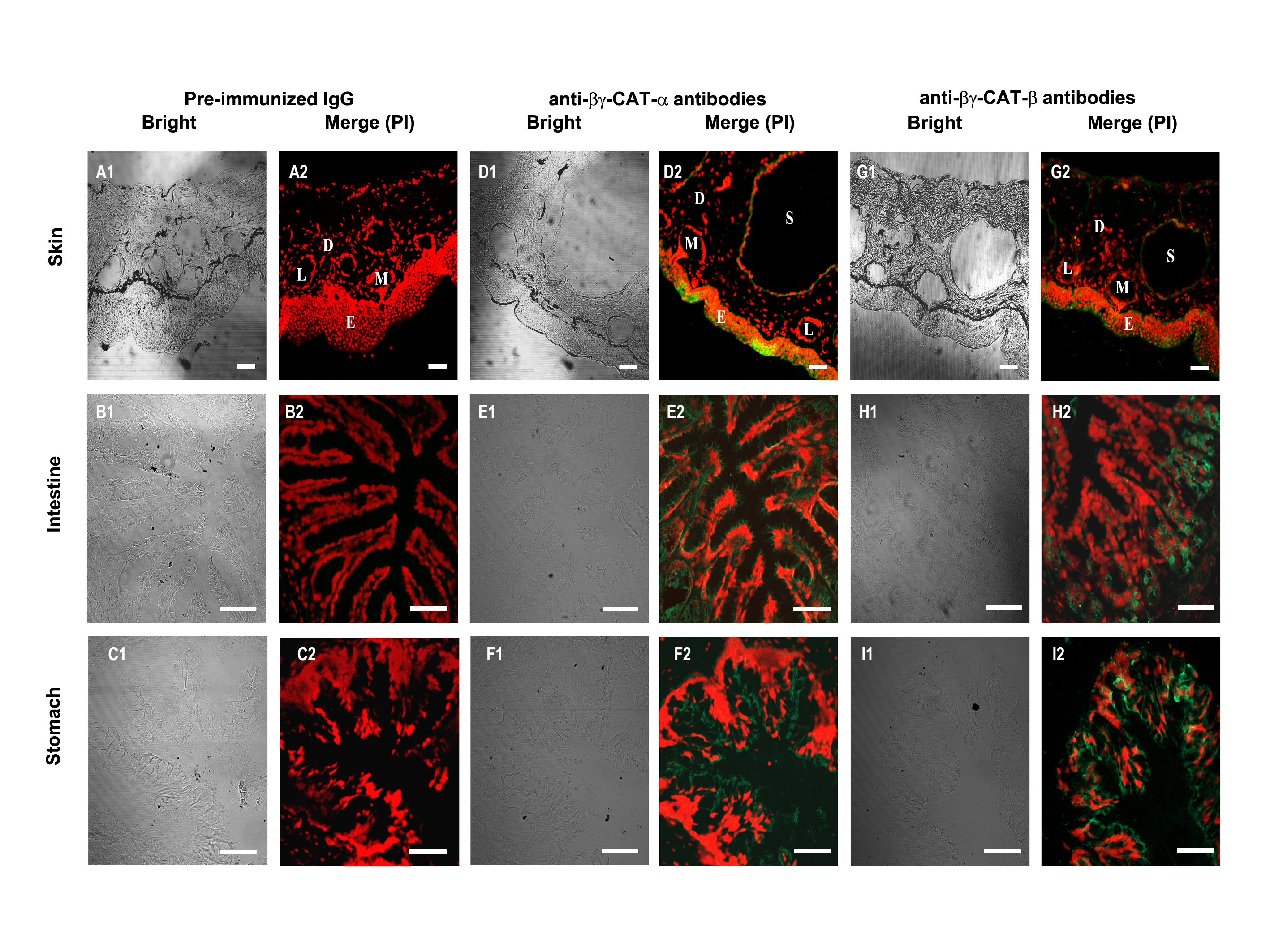

Supplement: Figure S2 — Distribution of betagamma-CAT and/or the homologues of its each subunit in frog B. maxima skin, intestine and stomach. Frog tissue cryostat sections were prepared as described in supporting information. Serial sections were stained with rabbit polyclonal antibodies against betagamma-CAT alpha-subunit (D1–F2), beta-subunit (G1–I2), respectively (green channel). Pre-immunized rabbit IgG (A1–C2) was used as control. Nucleus was counter-stained with PI (red channel). The same cryostat sections were observed by phase contrast in order to demonstrate morphology (A1, B1 and C1; D1, E1 and F1; G1, H1 and I1). Scale bars equal to 100 µm. In A2, D2 and G2, S, serous glands; L, lipid glands; M, mucous glands; D, dermis; E, epidermis. (6.41 MB TIF) [file pone.0001770.s003.tif]
